# A novel electrochemical IL-6 sensor based on Au nanoparticles-modified platinum carbon electrode

**DOI:** 10.3389/fbioe.2023.1128934

**Published:** 2023-02-16

**Authors:** Cai Wang, Dongyuan Xin, Qianwen Yue, Huiyu Wan, Qian Li, Ying Wang, Jingguo Wu

**Affiliations:** ^1^ The Second Affiliated Hospital, Shandong First Medical University & Shandong Academy of Medical Sciences, Taian, Shandong, China; ^2^ Binhai County People’s Hospital, Yancheng, Jiangsu, China; ^3^ Taishan Vocational College of Nursing, Taian, Shandong, China

**Keywords:** interleukin-6, electrochemical sensor, Au nanoparticles, antigen-antibody reaction, detection

## Abstract

**Introduction:** Interleukin-6 (IL-6) is a multifunctional polypeptide cytokine composed of two glycoprotein chains, which plays an important role in many cellular reactions, pathological processes, diagnosis and treatment of diseases and so on. The detection of IL-6 plays a promising role in the cognition of clinical diseases.

**Methods:** 4-mercaptobenzoic acid (4-MBA) was immobilized on the gold nanoparticles modified platinum carbon (PC) electrode with the linker IL-6 antibody, and finally formed an electrochemical sensor that specifically recognized IL-6. Through the highly specific antigen-antibody reaction, the IL-6 concentration of the samples to be detected. The performance of the sensor was studied by cyclic voltammetry (CV) and differential pulse voltammetry (DPV).

**Results:** The experimental results showed that the linear detection range of the sensor for IL-6 was 100 pg/mL–700 pg/mL and the detection limit was 3 pg/mL. In addition, the sensor had the advantages of high specificity, high sensitivity, high stability and reproducibility under the interference environment of bovine serum albumin (BSA), glutathione (GSH), glycine (Gly) and neuron specific enolase (NSE), which provided a prospect for specific antigen detection sensor.

**Discussion:** The prepared electrochemical sensor successfully detected the content of IL-6 in standard and biological samples, showing excellent detection performance. No significant difference was found between the detection results of the sensor and that of ELISA. The sensor showed a very broad prospect in the application and detection of clinical samples.

## 1 Introduction

Interleukin 6 (IL-6) is a multifunctional polypeptide cytokine composed of two glycoprotein chains. IL-6 promoted the proliferation of immune cells, cell differentiation and inhibited apoptosis and other functions. It also played a very important role in inflammatory response. In a variety of acute inflammatory reactions, IL-6 was produced rapidly and affectd the occurrence and development of the disease. According to the research and clinical findings, the concentration of IL-6 was closely related to the severity of inflammation. In addition, with the deepening of research, IL-6 played an important role in the occurrence, development, treatment and prognosis of tumors, metabolic diseases and other diseases (Tertiş et al., 2017; Russell et al., 2019; Khan et al., 2020; Chandra Barman et al., 2021).

In order to detect the content of IL-6 *in vivo* and *in vitro*, a variety of highly specific, sensitive and reliable detection techniques had been developed, such as enzyme-linked immunosorbent assay, colorimetry, photology, immunochromatography and so on (Gu et al., 2018; Saikrithika and Senthil Kumar, 2020; Chen et al., 2021; Xu et al., 2021). These methods had some disadvantages such as low sensitivity, tedious operation, poor reproducibility and so on (Jin and Maduraiveeran, 2017; Mihrican and Melike, 2018; McEachern et al., 2020; Rusheen et al., 2020). In recent years, electrochemical sensor had aroused great interest because of its fast response, high sensitivity and simplicity. It had been widely used in production and life. It was one of the most promising detection technologies (Li et al., 2014; Xu et al., 2017; Amiri et al., 2018; Lim and Bonanni, 2020; Balkourani et al., 2021).

**SCHEME 1:**
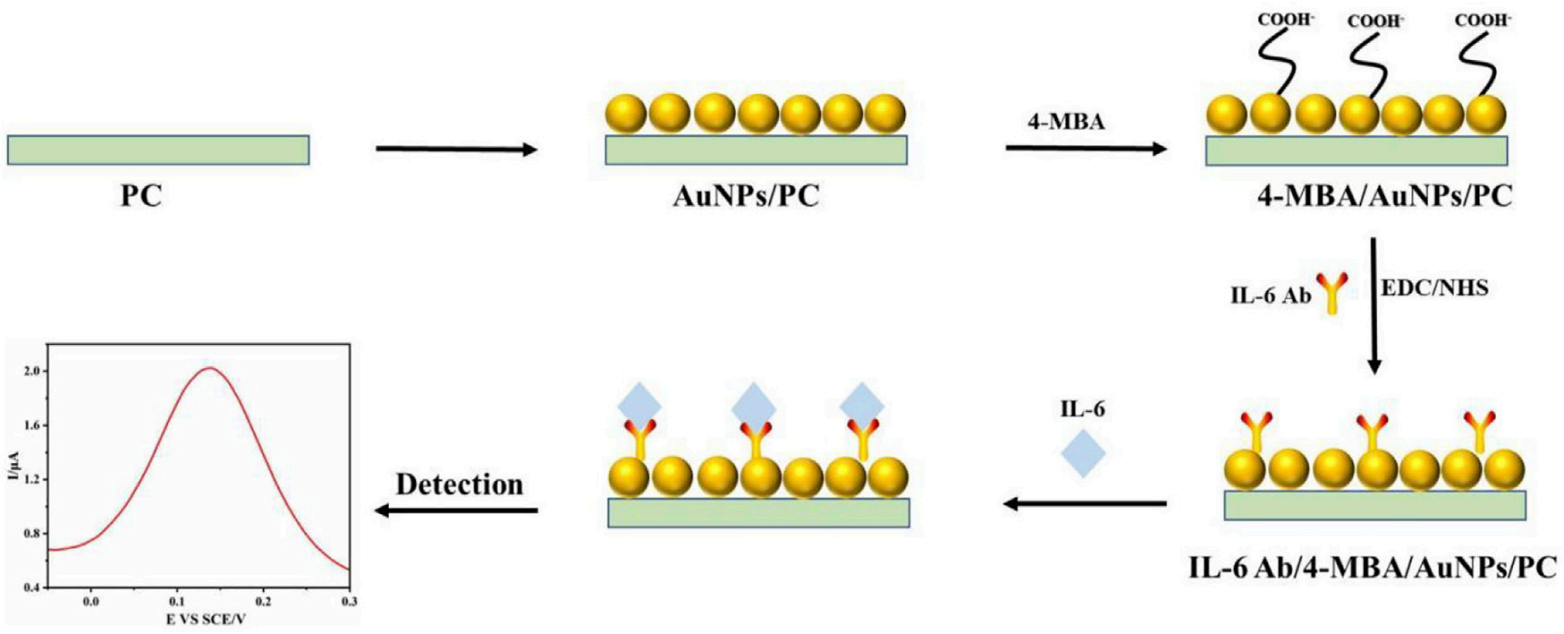
Preparation and schematic diagram of electrochemical sensor for specific detection of IL-6.

In this paper, in order to detect IL-6 in samples quickly, sensitively and accurately, we developed an electrochemical biosensor based on antigen-antibody specific reaction. As shown in Scheme 1, firstly, we prepared spherical Au nanoparticles (AuNPs) to modify the surface of platinum carbon electrode (PC) to facilitate the connection of signal molecule 4-mercaptobenzoic acid (4-MBA). In order to activate the carboxyl group of 4-MBA for better connection with IL-6 antibody (IL-6Ab), we used (1 (3-dimethylamino-propyl)-3-ethylcarbon diimide hydrochloride/N-hydroxysuccinimide (EDC/NHS) to play this role. Studying the performance of the sensor, we use cyclic voltammetry (CV), differential pulse voltammetry (DPV) and other methods. In addition, the sensor had the advantages of high specificity, high sensitivity, high stability and reproducibility under the interference environment of bovine serum albumin (BSA), glutathione (GSH), glycine (Gly) and neuron specific enolase (NSE), which provided a prospect for specific antigen detection sensor. Through the detection of IL-6 in standard solution, the experimental results showed that the linear detection range of the sensor for IL-6 was 100 pg/mL-700 pg/mL, and the detection limit was 3 pg/mL. We also detected the IL-6 content of clinical samples, showed a high consistency with the results of ELISA. For clinical and laboratory, the electrochemical sensor prepared by us has excellent performance such as high speed, convenience and high sensitivity, and has a very broad application prospect in real life and production.

## 2 Experimental section

### 2.1 Materials and instruments

(1 (3-dimethylamino-propyl) -3-ethylcarbon diimide hydrochloride/N-hydroxysuccinimide (EDC/NHS), 4-mercaptobenzoic acid (4-MBA) was purchased from Aladdin Industrial Co., Ltd. Chloroauric acid (HAuCl_4_), silver nitrate (AgNO_3_), hydrochloric acid (HCl), trisodium citrate (Tc), hydroquinone (Hq), potassium ferricyanide (potassium ferricyanide), potassium chloride (KCl), bovine serum albumin (BSA), glutathione (GSH) and glycine (Gly) were purchased from National Pharmaceutical Group Chemical Reagent Co., Ltd. IL-6 antibody, IL-6 antigen and neuron specific enolase (NSE) were purchased from Sigma Aldrich (Shanghai) Trading Co., Ltd. Ultra-pure water was filtered through Milli-Q reagent water system in the experiment. Electrochemical workstation (CHI660E), platinum carbon electrode, reference electrode and counter electrode were purchased from Shanghai Chenhua instrument Co., Ltd. It was purchased from Sedorius Scientific Instruments (Beijing) Co., Ltd. with PB21 acidity meter.

### 2.2 Preparation of Au nanoparticles

Selected 50 mL beaker, washed detergent and ultra-pure water for three times in sequence. Added 30 mL ultra-pure water to the beaker, boil, added 300 μL 1% HAuCl_4_ and 900 μL 1% Tc, stir, continued to heat until the solution turns wine red, and cool to room temperature to complete the preparation of gold seed solution. Selectd the 100 mL beaker, cleaned according to the above method, took 99 mL ultra-pure water to add the beaker, stir, added 1% HAuCl_4_ of 1 mL, 100 μL dilute HCl, gold seed solution of 1 mL, AgNO_3_ solution of 1 mL (10^–3^ mol/L), Hq solution of 4 mL (10^–3^ mol/L), stir until dark blue.

### 2.3 Construction of electrochemical sensor

The spherical AuNPs were modified on the PC electrode, and then 4-MBA was modified on the surface of the electrode after the complete reaction. In order to activate the carboxyl group of 4-MBA to facilitate closer connection with IL-6 antibodies, we used EDC/NHS to do this. After the full action of EDC/NHS, the IL-6 antibody was modified on the 4-MBA modified electrode, and finally the electrochemical sensor was prepared. All the reaction steps were carried out in a 37°C incubator.

### 2.4 Detection of IL-6 in standard sample

The standard solution of IL-6 antigen of known concentration was prepared with 0.1 mol/L PBS. Then it was added to the mixed solution of potassium ferricyanide (K₃[Fe(CN)₆]) and potassium chloride (KCl) (reaction system solution). The electrochemical sensor electrode was placed in the mixture, and the reference electrode and counter electrode were added at the same time. The correlation between CV and DPV was detected, and the linear relationship between concentration and peak current was obtained. The detection limit of the sensor was calculated. According to the linear relationship, the concentration of IL-6 in the sample solution to be tested was calculated.

## 3 Result and discussion

### 3.1 Preparation and characterization of AuNPs

**FIGURE 1:**
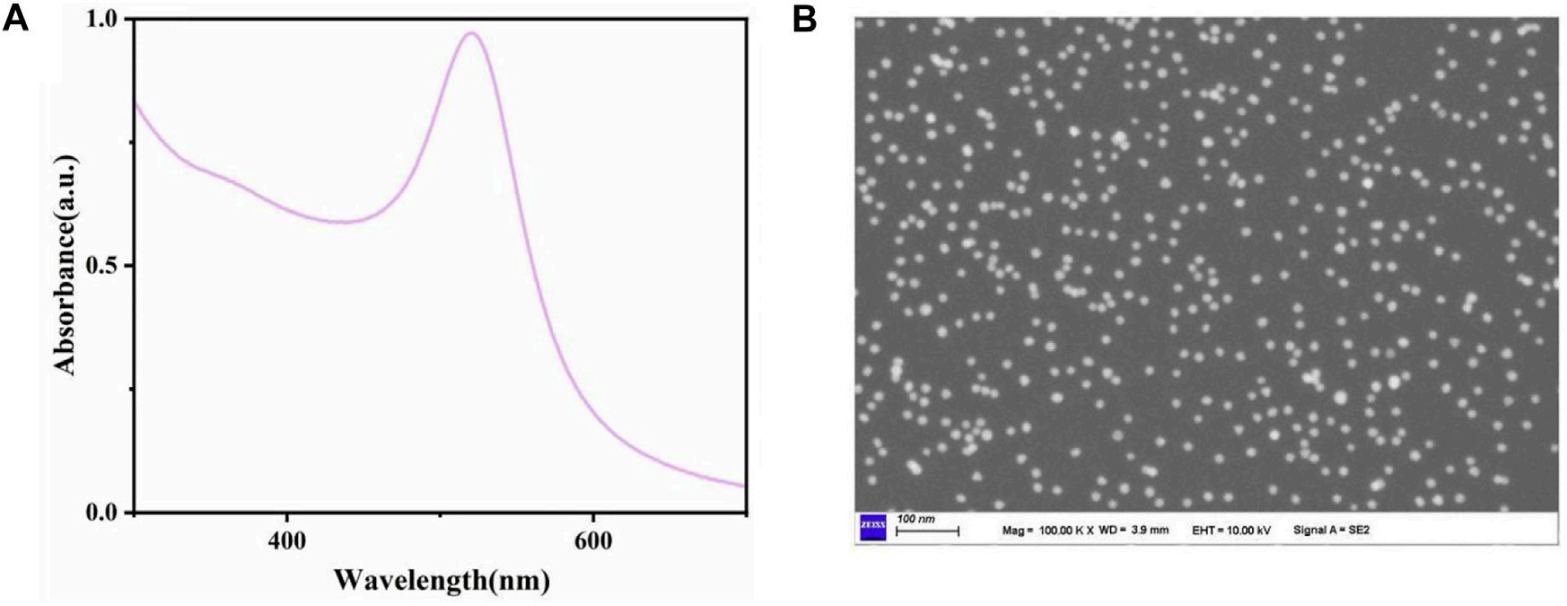
**(A)** The UV-vis absorption peak of AuNPs was about 520 nm. **(B)** SEM images of AuNPs.

The fabrication process of AuNPs was described above. In this part, we mainly introduced their characterization results. As shown in Figure 1A, the ultraviolet and visible light (UV-vis) absorption spectrum of the gold seed made of Venus was detected. The absorption peak of UV-vis of AuNPs was about 520 nm, which was consistent with the previous research. In addition, we used scanning electron microscope (SEM) to observe the nano-Venus particles, as shown in Figure 1B, the AuNPs was obviously angular, and its diameter was about 90 nm, which was in line with our experimental expectations.

### 3.2 Construction and characterization of the sensor

**FIGURE 2:**
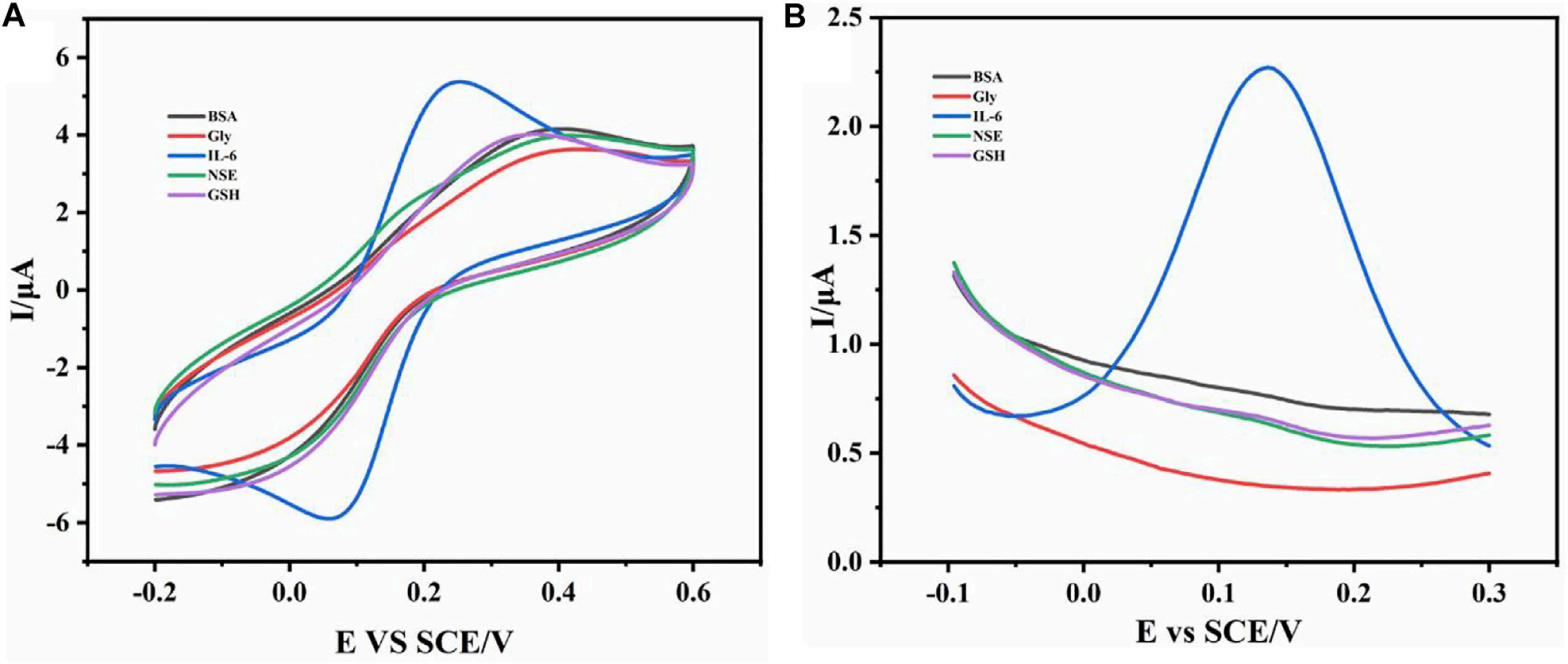
The CV curve **(A)** and DPV curve **(B)** of different substances (BSA, Gly, GSH, NSE, and IL-6) were added.

According to the above methods, we prepared electrochemical sensors for specific recognition of IL-6. In order to verify the specific recognition function of the sensor, we used CV and DPV to detect the sensor. As shown in Figure 2A, we successively added BSA, Gly, GSH, NSE, and IL-6 to the reaction solution and detected it by electrochemical sensor. The experimental results showed that the clear redox peak was only seen in the cyclic voltammogram of the reaction solution with IL-6, which indicated that the sensor recognizes and specifically binds IL-6 in the solution. In addition, in the differential pulse voltammogram (Figure 2B), we also found that only the DPV curve of the solution added with IL-6 had a peak, which further proved the specific recognition and binding function of the sensor to IL-6. Through the atlas results of CV and DPV, we determined that the IL-6 electrochemical sensor had the ability to specifically recognize and bind IL-6, which indicated that our sensor was constructed successfully.

**FIGURE 3:**
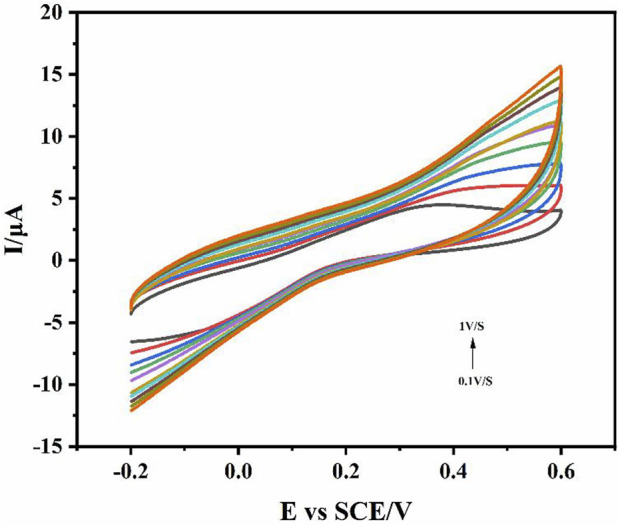
The CV curves of different scanning rates in the reaction solution of standard concentration of IL-6. Scan rate 0.1V/S-1V/S.

In order to evaluate the kinetic characteristics of the sensor, the cyclic voltammogram (Figure 3) of the sensor electrode in the reaction solution of standard concentration of IL-6 was recorded at different scanning rates. The oxidation peak current and reduction peak current increased with the change of scanning speed (0.1V/S-1V/S). Through the cyclic voltammogram, it was found that the peak potential did not change with the change of the scanning rate, which indicated that the redox state of the IL-6 electrochemical sensor is reversible, and the redox center was located on the surface of the electrode.

### 3.3 Effects of sensors in different pH reactants

**FIGURE 4:**
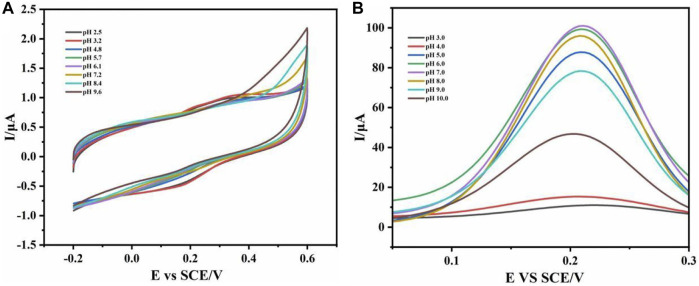
CV curve **(A)** and DPV curve **(B)** were detected in different pH reaction solution systems.

To explore the best working pH of the electrochemical sensor to allow it to successfully recognize IL-6 antigen in the pH environment of the real sample, we studied its performance in different pH reaction systems. The detection results of CV and DPV in different pH environments (Figure 4) showed that the sensor works better in neutral environment. As shown in Figure 4A, CV was detected in the reaction solution of pH 2.5–9.6. The results showed that the redox peak changed obviously in the reaction solution of pH 7.2. Compared with the test results of CV, the test results of DPV (Figure 4B) were more obvious. In the reaction solution of pH 7.0, the peak current reached the maximum, indicated that the sensor may be the best working environment in the neutral solution, which was close to the pH value of human blood and in line with the expectation of clinical transformation.

### 3.4 Detection of IL-6

**FIGURE 5:**
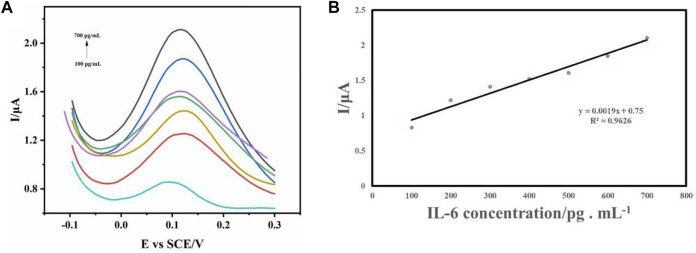
**(A)** DPV curves of sensor electrodes with different concentrations of IL-6 were detected. **(B)** The relationship between peak current and concentration.

To evaluate the electrochemical analysis performance of IL-6 sensor, IL-6 detection was carried out under the above optimized conditions. Differential pulse voltammetry (DPV) had been recognized as an important tool for rapid electrochemical detection and analysis because of its high current sensitivity (Ou et al., 2007; He et al., 2008; Cevik et al., 2016; Zhang et al., 2017). Therefore, in order to analyze the sensitivity of the sensor and its relationship with concentration, we measured the DPV curves (Figure 5A) of many standard solutions with known IL-6 concentrations. As shown in Figure 5A, the oxidation peak current of the sensor increased gradually with the increase of IL-6 concentration, and the position of the oxidation peak was stable at about 0.12 V. There was a good linear relationship between oxidation peak current and concentration (I (μA) = 0.0019C_IL-6_ (ng.mL^−1^) + 0.75, *R* ^2^ = 0.9626), which was obtained by IL-6 of 100 pg/mL-700 pg/mL concentration (Figure 5B). According to these relationships, we preliminarily calculated that the detection of the sensor was 3 pg/mL.

### 3.5 Reproductivity assay

**FIGURE 6:**
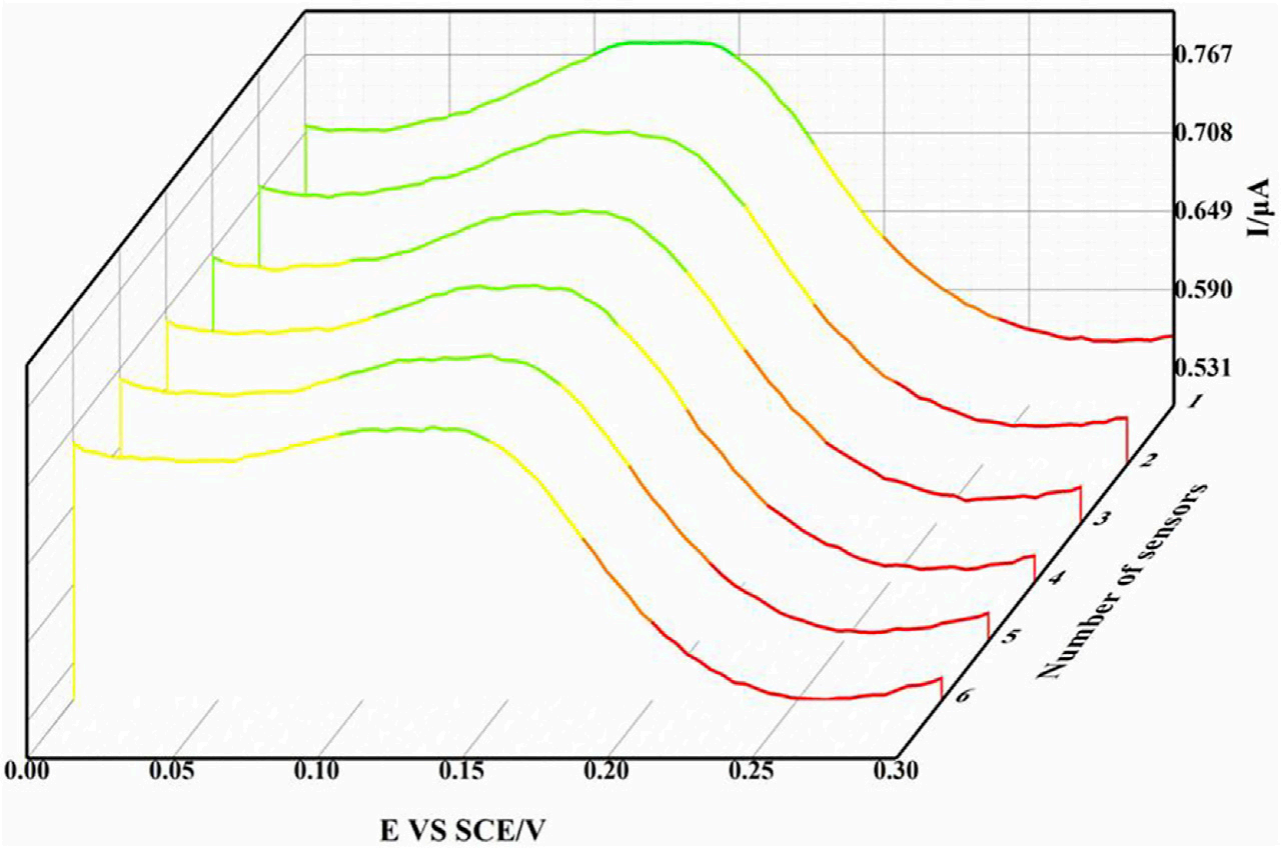
Three-dimensional waterfall diagram of DPV curve of six sensors.

In order to study the reproducibility of IL-6 electrochemical sensors, we prepared six sensors to detect DPV in the same detection environment under the same conditions. As shown in Figure 6, a three-dimensional waterfall map was made based on the DPV data of six electrochemical sensors. From the graphic point of view, the waveforms of the six sensors had excellent consistency. At about 0.12 V, the relative standard deviation of the peak current was 2.35%. This showed that the sensor had excellent reproducibility and had a broad prospect in the detection of clinical samples.

### 3.6 Real sample detection

**FIGURE 7:**
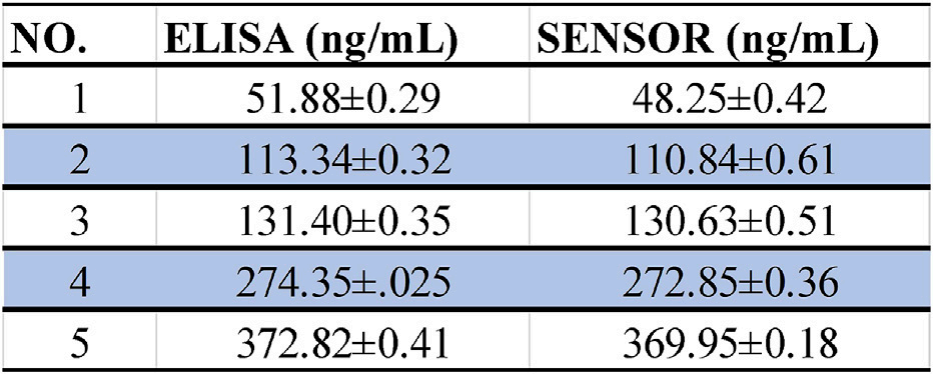
Statistics of ELISA and sensor detection results of the same sample.

To further evaluate the prospect of the sensor in the detection of clinical real samples, we collected serum samples from patients with spinal cord injury. In order to compare its accuracy, the test results of ELISA WEre used as reference data. As shown in Figure 7, the blood samples of the same sample were detected by ELISA and electrochemical sensor respectively, and the results showed high consistency. The experimental results showed that the prepared electrochemical sensor had good accuracy, and the operation was more simple and fast than ELISA. Moreover, we also compared other technical data for the detection of IL6 (Table1). The electrochemical sensor also showed better detection performance, which had a very broad prospect in clinical application.

**TABLE 1:** Different methods to detect IL-6.

NO.	Test method	Detection limits	References
1	SERS,ICA	7.897 pg/mL	Xia et al. (2020)
2	SERS	1.6 pg/mL	Wang et al. (2021)
3	Optics	0.25 nM	Anastasopoulou et al. (2018)
4	SPFS	2 pg/mL	Toma and Tawa, (2016)
5	Fiber-optic	5p.m.	Kapoor and Wang, (2009)
6	Impedimetric aptasensor	1.6 pg/mL	Tertis et al. (2019)
7	Impedimetric immunosensor	6 fg/mL	Aydin, (2020)
8	Photoelectrochemical	0.38 pg/mL	Fan et al. (2014)
9	Electrochemical immunosensor	7 fg/mL	Cao et al. (2020)
10	Electrochemical immunosensor	0.059 pg/mL	Lou et al. (2014)
11	Fibre sensor	0.1 pg/mL	Zhang et al. (2018)
12	SERS	1 pg/mL	Wang et al. (2013)
13	Immunofluorescence	0.5 pg/mL	Shakeri et al. (2020)

SERS, surface enhanced Raman spectroscopy; ICA, immunochromatographic assay; SPFS, surface plasmon enhanced fluorescence spectroscopy.

## 4 Conclusion

A novel electrochemical sensor for the detection of IL-6 was prepared by successively modifying the surface of the electrode with AuNPs,4-MBA,EDC/NHS,IL-6 antibody. Through the experimental results of CV and DPV, it was found that the electrochemical sensor had the advantages of high specificity, high sensitivity, high stability and reproducibility. The oxidation peak current of DPV showed a good linear relationship with the concentration of IL-6, and the detection limit was 3 pg/mL. Combined with the detection results of real samples, the prepared electrochemical sensor showed a very broad application prospect.

## Data Availability

The raw data supporting the conclusion of this article will be made available by the authors, without undue reservation.
